# GERONTOLOGY AND GERIATRICS PROGRAMS: OBSERVATIONS FROM THE US DATABASE AT THE UNIVERSITY OF NEBRASKA

**DOI:** 10.1093/geroni/igae098.1035

**Published:** 2024-12-31

**Authors:** Mark Staley, Julie Masters

**Affiliations:** University of Nebraska Lincoln, Lincoln, Nebraska, United States; University of Nebraska, Lincoln, Nebraska, United States

## Abstract

Since its inception in 2022, the United States Gerontology and Geriatrics programs database developed and housed on the University of Nebraska-Omaha (UNO) Department of Gerontology website serves as a repository of educational programs at the undergraduate, graduate, and medical fellowship levels. Established to provide an easily accessible tally of programming and a resource for students, faculty, and professionals, the database is also intended to promote gerontological and geriatric programs across the U.S. Over time, the database has expanded to include Research Institutes, Nurse Practitioner programs, Medical Fellowships, Social Work programs, and designations including Age Friendly Universities (AFU) and Program of Merit (POM). With growth, the database is reviewed for accuracy, additions, and deletions. A troubling trend, however, has been the pausing and/or elimination of once healthy programs. In addition to presenting an overview of these observations (e.g., number of programs, changes), this session explores reasons for closure and what lessons can be learned for those still in operation.

